# Valine-Induced Isoleucine Starvation in *Escherichia coli* K-12 Studied by Spike-In Normalized RNA Sequencing

**DOI:** 10.3389/fgene.2020.00144

**Published:** 2020-03-05

**Authors:** Bertil Gummesson, Shiraz Ali Shah, Alexander Skov Borum, Mathias Fessler, Namiko Mitarai, Michael Askvad Sørensen, Sine Lo Svenningsen

**Affiliations:** ^1^ Department of Biology, University of Copenhagen, Copenhagen, Denmark; ^2^ Niels Bohr Institute, University of Copenhagen, Copenhagen, Denmark

**Keywords:** stringent response, deep RNA sequencing, whole-cell spike-in normalization, ribosomal RNA degradation, transcriptome, ppGpp, gene expression

## Abstract

*Escherichia coli* cells respond to a period of famine by globally reorganizing their gene expression. The changes are known as the stringent response, which is orchestrated by the alarmone ppGpp that binds directly to RNA polymerase. The resulting changes in gene expression are particularly well studied in the case of amino acid starvation. We used deep RNA sequencing in combination with spike-in cells to measure global changes in the transcriptome after valine-induced isoleucine starvation of a standard *E. coli* K12 strain. Owing to the whole-cell spike-in method that eliminates variations in RNA extraction efficiency between samples, we show that ribosomal RNA levels are reduced during isoleucine starvation and we quantify how the change in cellular RNA content affects estimates of gene regulation. Specifically, we show that standard data normalization relying on sample sequencing depth underestimates the number of down-regulated genes in the stringent response and overestimates the number of up-regulated genes by approximately 40%. The whole-cell spike-in method also made it possible to quantify how rapidly the pool of total messenger RNA (mRNA) decreases upon amino acid starvation. A principal component analysis showed that the first two components together described 69% of the variability of the data, underlining that large and highly coordinated regulons are at play in the stringent response. The induction of starvation by sudden addition of high valine concentrations provoked prominent regulatory responses outside of the expected ppGpp, RpoS, and Lrp regulons. This underlines the notion that with the high resolution possible in deep RNA sequencing analysis, any different starvation method (e.g., nitrogen-deprivation, removal of an amino acid from an auxotroph strain, or valine addition to *E. coli* K12 strains) will produce measurable variations in the stress response produced by the cells to cope with the specific treatment.

## Introduction

During stress conditions, cells of *Escherichia coli* (*E. coli*) impose dramatic changes in their transcriptional profile and proteome to combat stressors. The cells ensure that genes important to overcome the stress are turned on and other redundant and energy-demanding gene products, such as genes of the protein synthesis machinery [*i.e.,* those encoding the ribosomes, transfer RNAs (tRNAs) and factors required for translation] are down-regulated. The rapid re-orchestration of the transcriptome in *E. coli* occurs on the timescale of a few minutes, and is aided by the small molecules guanosine tetra- and pentaphosphate, herein collectively referred to as ppGpp. This physiological response is called the stringent response (Ryals et al., 1982; Cashel et al., 1996) and has become a model system for studies of bacterial stress responses. Together with the protein DksA, ppGpp binds two sites on RNA polymerase, which affects promoter selectivity and reduces the ribosomal RNA (rRNA) promoter clearing rates (Artsimovitch et al., 2004; Gummesson et al., 2013; Ross et al., 2016). The nucleotide ppGpp is produced when amino acids become limiting and upon starvation for many different kinds of nutrients as well as by other circumstances restricting growth (Cashel et al., 1996). In *E. coli*, the synthesis of ppGpp is mediated by two related proteins, RelA and SpoT; each requiring different signals for activation. The RelA protein is associated with uncharged tRNA and the synthesis of ppGpp is triggered when the translating ribosome binds a RelA-tRNA complex at the starving A-site codon (Haseltine and Block, 1973; Winther et al., 2018). The SpoT protein is bi-functional; besides synthesizing ppGpp, SpoT can hydrolyse ppGpp to guanosine diphosphate and pyrophosphate (Murray and Bremer, 1996), thus allowing a way out of stringency when conditions allow.

The global effect of ppGpp on transcription has previously been studied upon starvation for the amino acid serine (Durfee et al., 2008) or isoleucine (Traxler et al., 2008; Traxler et al., 2011). These studies have in common that they utilized the well-established expression microarrays as their read out, the best technology available for genome-wide analysis at the time. However, the much more sensitive technique of deep RNA sequencing (RNAseq) has emerged as a standard method to measure globally the relative abundance of RNA species in the cell, and offers a superior dynamic range for measuring variability in the levels of expressed transcripts (Wang et al., 2009; Croucher and Thomson, 2010). The effects of ppGpp on global transcriptional regulation without concomitant starvation has recently been studied using RNAseq, and resulted in a substantial expansion of the genes that can be assigned to the ppGpp-controlled regulon (Sanchez-Vazquez et al, 2019).

The long-lived house-keeping RNAs, rRNA, and tRNA, constitute the vast majority of the total RNA in the cell (>95%) (Bremer and Dennis, 1996). For this reason, rRNA and sometimes tRNA are generally removed prior to RNAseq, or not included on microarrays, unless they are the specific focus of the study. One goal of our work was to obtain data on the response to amino acid starvation in *E. coli* that includes the changes in the whole transcriptome, including the most abundant RNAs, and to analyze how inclusion of all RNA may enhance the current understanding of the well-studied stringent response. In connection with this goal came the need to quantify transcripts without making assumptions about the total RNA content of the cells before and after starvation. Typical transcriptome analyses, whether done by microarray or RNAseq, rely on the assumption that the total amount of RNA is constant across different sample conditions. However, while rRNA and tRNAs are generally believed to be stable during exponential growth (Baracchini and Bremer, 1987), the familiar way of thinking of these RNAs as stable in an absolute sense has been questioned for some time (Deutscher, 2003). Our previous work shows that a substantial fraction of the tRNA and rRNA pools in the cell is rapidly degraded upon amino acid starvation (Svenningsen et al., 2017; Fessler et al., 2020), suggesting that the total RNA content of *E. coli* cells may decrease appreciably under this condition. Given the global changes in gene expression and the possibility that total RNA levels may decrease upon amino acid starvation we reasoned that a normalization method that is independent of any assumptions about cellular RNA content would be important for accurate detection of gene expression changes during the stringent response. Therefore, we chose to normalize the sample sequencing reads using a spike-in culture for reference. Spike-in, in the form of *in vitro* synthesized RNA, has been used in many experiments for normalization of transcriptional activity (see e.g., Schena et al., 1995; Bartholomäus et al., 2016; Gorochowski et al., 2019) and to verify the accuracy of RNA preparation protocols (see e.g., Jones et al., 2015; Ju et al., 2019). However, *in-vitro*-transcribed spike-in RNA is added after the extraction of the experimental RNA and quantification of transcription rates assume an equally efficient extraction of RNA from each sample (Gorochowski et al., 2019). Or, if the spike-in transcripts are added per mass of RNA in each sample, the underlying assumption is that cells contain equal amounts of RNA at the different conditions. The whole-cell spike-in approach is often used in microbiome studies to quantify cell numbers (Hornung et al., 2019) but has not, to our knowledge, been used outside our research group for quantification of RNA (Svenningsen et al., 2017). The benefit of the whole-cell spike-in approach we use here is that it allows normalization directly to the concentration of bacteria in each sample [as measured by optical density (OD)], without making any assumptions about the RNA content of the cells. For comparison, we also normalized our data set using the conventional approach of normalizing the data based on the sequencing depth obtained for each sample. The analysis of the transcriptome of isoleucine-starved cells normalized by the two methods reveal that the regulon responding negatively to starvation is much larger than what is detected using a conventionally normalized RNAseq transcriptome, and the regulon responding positively is correspondingly smaller. This observation relates to a greater turnover of total RNA in starved cells than previously anticipated, and the spike-in approach enabled us to quantify the loss of rRNAs and total messenger RNA (mRNA) during starvation relative to the levels during steady-state growth. Furthermore, principal component analysis of the stringent response transcriptome reveals two predominant temporal gene profiles that are enriched for classes of genes with related biological functions. Finally, it was evident that isoleucine starvation induced by L-valine has transcriptional consequences that are separate from the general stringent response of amino-acid-starved cells controlled by ppGpp.

## Materials and Methods

### Strains, Media, and Growth Condition

The wild-type strain *E. coli* K-12 MAS1081 (MG1655 *rph^+^ gatC^+^ glpR^+^*) were grown in flasks at 37°C at 200 rpm in morpholinepropanesulfonic acid (MOPS) minimal medium (Neidhardt et al., 1974) supplemented with 0.2% glucose. Cell growth was monitored spectrophotometrically by optical density at 436 nm (OD_436_) and cultures were grown for at least nine generations in exponential phase before sampling. Isoleucine starvation was induced by adding L-valine to a final concentration of 400 µg/ml (Leavitt and Umbarger, 1962). The small RNA (sRNA) *qrr2* from *Vibrio cholerae* was cloned downstream of the T7 promoter in the vector pET11a (XbaI/Bpu1102I) and transformed into *E. coli* BL21 (DE3) to yield the spike-in strain BKG3; 100 µg/ml ampicillin was used to maintain the plasmid and the expression of Qrr2 was induced with 1 mM isopropyl β-D-1-thiogalactopyranoside (IPTG). Rifampicin was added to a final concentration of 300 µg/ml immediately after the last isoleucine starvation sample to block transcription initiation.

### Spike-In and RNA Extraction

To preserve cellular RNA, bacterial culture samples were harvested by mixing with 1/6 vol of a stop-solution composed of 5% water-saturated phenol in ethanol at 0°C (Bernstein et al., 2002). All samples were kept at 0°C until the final sample had been harvested. Prior to total RNA extraction, a volume of spike-in culture corresponding to 1% of the experimental culture was added to each sample, based on sample OD. The volume of spike-in cell culture used was calculated according to Equation 1 (as described in Stenum et al., 2017).

(1) $$V_{spike-in}=\frac{0.01*V_{sample}*OD_{sample}}{OD_{spike-in}}$$

RNA was isolated using a hot phenol extraction method. Briefly, cell pellets were mixed with resuspension solution (0.3 M sucrose, 0.01 M sodium acetate pH 4.5, 0°C), then with lysis solution [2% sodium dodecyl sulfate (SDS), 0.01 M sodium acetate pH 4.5] and finally with hot acidic phenol [pH 4.3, 65°C (Fisher BioReagents)]. The mixture was snap-freezed in liquid nitrogen and centrifuged, and the aqueous phase was re-extracted by phenol (65°C) and frozen in liquid nitrogen one more time. RNA was precipitated with 2.5 vol ethanol and 0.1 vol sodium acetate (3M, pH 4.7) at −80°C overnight. Precipitated RNA was pelleted, washed with 70% ethanol, and re-suspended in nuclease-free H_2_O. The remaining DNA was removed by DNaseI treatment (Roche), according to the manufacturers manual. RNA integrity (16S and 23S rRNA) was verified by agarose gel electrophoresis.

### Northern Blot

An aliquot of total RNA was mixed with 3 vol loading dye (8 M urea, 6% formaldehyde, bromophenol blue) and fractionated by electrophoresis through a 1% MOPS-buffered agarose gel prepared with 6% formaldehyde. The RNA was transferred from the gel onto a Hybond-N+ membrane by capillary transfer overnight and was fixed to the membrane by 0.12 J/cm^2^ of UV light in a Hoefer UVC 500 UV crosslinker. Membranes were pre-hybridized for one hour at 42°C in 6 ml hybridization solution [0.09 M NaCl, 0.05 M NaH_2_PO_4_ (pH 7.7), 5 mM ethylenediaminetetraacetic acid (EDTA), 5x Denhardt's solution, 0.5% (w/v) SDS, 100 mg/ml sheared, denatured herring sperm DNA]. Hybridization of the immobilized RNA was performed at 42°C overnight in the same solution as above with 40 pmol ^32^P 5'end-labeled oligo-DNA probe (γ-[^32^P]-ATP; PerkinElmer). DNA-oligos used were complementary to a sequence in the 5S rRNA, 16S rRNA, 23S rRNA, or Qrr2, probe sequences are listed in **Supplementary Table S11**. Membranes were washed several times in 0.3 M NaCl, 30 mM sodium citrate, 0.1% SDS at room temperature prior to exposure to a phosphor-imaging screen. The radioactivity present in specific bands was measured on a Typhoon phosphor Imager FLA7000 (GE Healthcare) at 100 microns. Membranes were stripped of hybridized probes with 90–95°C stripping buffer (0.1% SDS, 18 mM NaCl, 1 mM NaH_2_PO_4_, 0.1 mM EDTA) under shaking until no more radioactivity could be detected on the blot by a Geiger-Müller tube. The program ImageQuant TL 8.2 was used to quantify each band on the phosphor-imaging screen. The quantified intensity on each rRNA band were then divided with the values from Qrr2 in the same lane and this ratio is plotted relative to the three samples harvested immediately before inducing starvation.

### Quantitative Reverse Transcription PCR

First-strand complementary DNA (cDNA) was reverse transcribed from 1 μg of total RNA with Thermo Scientific RevertAid RT Kit (#K1691) using the supplied random hexamer primers. As control for genomic DNA contamination, a reaction with no reverse transcriptase was included for each sample (RT-). A 1/10,000 to 1/25 fraction of the total synthesized cDNA was combined with SsoAdvanced Universal SYBR Green Supermix (Bio-Rad) and analyzed in triplicate by quantitative reverse transcribed PCR (qRT-PCR) using the QuantStudio 3 system (Applied Biosystems). Thermal cycling conditions used were 95°C for 30 s followed by 40 cycles of 95°C for 15 s, 60°C for 1 min. A final melting-curve cycle was performed to check for amplification artifacts starting at 95° for 15 s, 60° for 1 min, followed by a dissociation step to 95°C with 0.15°C/s increments. The relative levels of RNA is calculated as the signal ratio between the target transcript and one of the reference genes from the spike-in plasmid, namely *bla*, using the formula: 2^−(^ΔΔ*^C^* ^T)^ where ΔΔ*C*_T_ = (*C*_T,target_−*C*_T,_ *_bla_*)_time_ *_x_*−(*C*_T,target_−*C*_T,_ *_bla_*)_time__0__(steady_ _state),_ as previously described (Livak and Schmittgen, 2001). Primer sequences for target genes and control gene are listed in **Supplementary Table S11**.

### RNA Sequencing and Data Analysis

The RNA used for RNAseq was harvested, spiked-in, and extracted as described above; 1–1.5 µg of total RNA from each sample was sent to the GATC BIOTECH facility, European Genome and Diagnostics Centre, Konstanz, Germany for library preparation and RNA sequencing. RNA quality was assessed using an Agilent 2100 Bioanalyzer/Advanced Analytical Technologies Fragment Analyzer. Strand-specific cDNA libraries were prepared according to Illumina's protocols without prior rRNA depletion. RNAseq experiments were performed on an Illumina HiSeq using a paired-end read length of 2x50 bp. Twenty-two to 29 million paired-reads were obtained per sample. GATC BIOTECH initially processed the raw read files, removing adapters prior to delivery. Then the files were uploaded to the Galaxy web platform and we used the public server at usegalaxy.org to analyze the data (Afgan et al., 2018). The files were checked using fastQC^1^. The reads were then aligned to *Escherichia coli* str. K-12 substr. MG1655 (RefSeq NC_U00096.3) using bwa-aln (version 0.7.15.2 with default parameters) (Li and Durbin, 2009). Reads were counted using htseq-count (version 0.6.1p1) (Anders et al., 2015). In parallel, the reads were aligned (bwa-aln) to the reference sequence of the plasmid harboring the spike-in genes and raw read counts mapping to three features (*qrr2, bla,* and antisense-*lacI*), and counted using htseq-count and summed to give the plasmid spike-in reads for a given sample. Raw read counts were then normalized to gene size prior to normalization to spike-in reads to give RPKSP, Reads Per Kilobase of gene per 10 kilobase of spike-in as shown in Equation 2.

(2) $$RPKSP=\frac{\left(Gene-specific\;Reads\;Per\;Kilobase\right)}{\left(Spike-in\;Reads\;Per\;Kilobase/10.000\right)}$$

We emphasize that the order in which the raw reads were aligned to the *E. coli* chromosome and to the spike-in plasmid did not change the results. Specifically, the same results were obtained when the raw reads were separately aligned to the plasmid and the chromosome as when the alignment was carried out sequentially (i.e., reads were first aligned to the chromosome and remaining reads were aligned to the plasmid).

The raw read counts were also normalized according to the standard method to give RPKM, Reads Per Kilobase Million as shown in Equation 3.

(3) $$RPKM=\frac{\left(Gene-specific\;Reads\;Per\;Millionreads\right)}{\left(Size\;of\;Specific\;gene\left(kb\right)\right)}$$

High-throughput sequencing data has been deposited in NCBI's Gene Expression Omnibus (Edgar et al., 2002) and are accessible through GEO Series accession number GSE136753**** ^2^. ****

### Transcriptome Data Filtering

We initially applied some filtering of the normalized transcriptomic data (RPKSP and RPKM) in order to quantify the magnitude of fold differences in transcriptional regulation upon starvation for isoleucine. **i**) All genes that were neither sequenced in steady state nor in starvation were filtered out (50 genes). **ii**) Transcripts in the triplicate steady-state samples that either had low average normalized reads or no reads in combination with either low average normalized reads or no reads in the four starvation samples were filtered out (112 genes). These transcripts did not yield any computable fold differences between steady-state growth and starvation. One feature, the gene *lacI*, was present on both the *E. coli* chromosome and the spike-in plasmid. *lacI* was therefore excluded from our analysis. A third filtering step was applied in the comparison of fold differences between RPKM and RPKSP normalization at 10 and 80 min starvation, **iii**) genes where the fold change at both the 10 and 80 min time points relative to the steady-state average could not be calculated due to a lack of coverage were omitted (60 genes). In total 4,048 transcripts were assessed, i.e., 95% of the annotated genes in the *Escherichia coli* str. K-12 substr. MG1655 (RefSeq NC_U00096.3) reference genome. The average standard deviation between the three steady-state measurements of each of the 4,048 transcripts was 25%. The normalized sequencing reads, including omitted genes, are reported in **Supplementary Data Sheet 1** (RPKSP) and **Supplementary Data Sheet 2** (RPKM). For the analysis of the variance among steady-state samples as a function of gene length (**Supplementary Table S2**), we also applied a filter; the analysis was restricted to only consider genes where at least two of the triplicate steady-state samples had detectable transcripts. This yielded 3.979 transcripts for analysis.

### Principal Component Analysis

Principal component analysis (PCA) (Abdi and Williams, 2010) was performed on RPKSP-normalized reads of the steady-state samples and the starvation time series in **Supplementary Data Sheet 1**. In order to focus on the temporal profile of expression changes and not the absolute expression level, the number of reads for each data point were normalized to the average number of reads mapped to the corresponding gene for the seven time points. PCA was performed and visualized in MATLAB (MATLAB, Release R2016b). PC1 and PC2 captured 48 and 21% of the variability of the data, respectively. PC3 captured 9% of the variability, but the PC3 vector showed large variability among the three steady-state samples, indicating that it captured a trend that is due to sampling error. Therefore, we focused on the first two principal components. For the enrichment analysis, the Enrichment tool in the SmartTable of the EcoCyc webserver (Karp et al., 2014; Keseler et al., 2017) was used with the options of “Fisher Exact” and “Benjamini-Hochberg Correction” on “Biological Process” gene ontology terms.

### Ecocyc Omics Dashboard Tool

Genes in **Supplementary Data Sheet 1** and the log_2_-fold induction ratios of the data points in the starvation time series were imported as a SmartTable in Ecocyc (Karp et al., 2014; Keseler et al., 2017) and analyzed using the Omics Dashboard Tool (Paley et al., 2017). The Dashboard Biosynthesis shown in **Figure 8A** was modified to only show the seven largest sub-systems of biosynthetic genes. In addition, the group of aminoacyl-tRNA synthetases was added manually by curating and extracting the relevant genes from the Biosynthesis sub-system “Others.” Genes belonging to the arginine biosynthesis sub-system were exported and their induction ratio at the 5 min time point after starvation are shown alongside data on the same genes extracted from the dataset published by Sanchez-Vazquez and co-workers (Sanchez-Vazquez et al., 2019).

## Results

### Experimental Approach and Provoking Amino Acid Starvation

**Figure 1:**
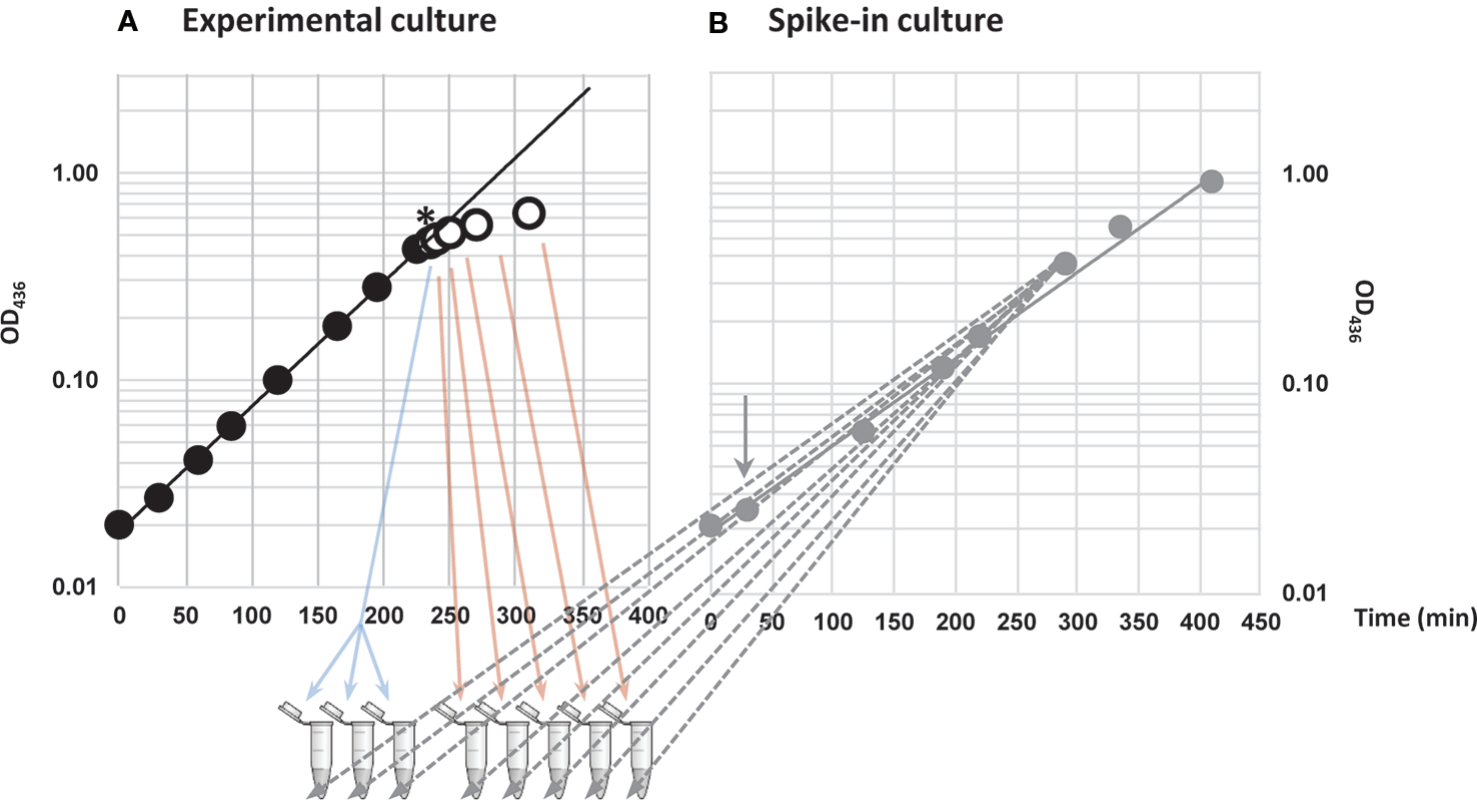
Provoking amino acid starvation and addition of spike-in cells to samples. **(A)** Cells were grown in steady state (closed circles) before induction of isoleucine starvation by addition of 400 μg/ml L-valine (denoted by *). Three steady-state samples (blue arrows) were harvested as reference immediately before addition of L-valine and five samples in total were harvested during starvation up to 80 min after the addition of L-valine (open circles and red arrows). **(B)** In parallel, an *E. coli* strain that carried an inducible plasmid expressing a *Vibrio cholerae* small RNA (sRNA) (*qrr2*), antisense-*lacI* and an antibiotic marker (*bla*) (gray closed circles) was grown as a spike-in culture. Addition of inducer (1 mM IPTG) is indicated with a gray arrow. The samples collected in **(A)** were spiked-in with 1% optical density (OD) units of the spike-in culture of cells harboring the plasmid (gray dashed arrows).

To evoke amino acid starvation in cultures of *E. coli* K-12, we grew MAS1081 (MG1655; *rph* ^+^ *gatC* ^+^ *glpR* ^+^) in MOPS-buffered minimal medium supplemented with 0.2% glucose and starved for the amino acid isoleucine by adding excess L-valine. The K-12 strain of *E. coli* harbors a frameshift mutation in *ilvGM*, inactivating one of three isozymes in the valine and isoleucine biosynthetic pathways, while the other two isozymes, *ilvBN* and *ilvIH*, are susceptible to feedback inhibition by L-valine (Valle et al., 2008). High concentrations of L-valine therefore renders *E. coli* K-12 auxotrophic for isoleucine (Leavitt and Umbarger, 1962). Three samples were collected during steady-state growth immediately before starvation and five samples in total were collected in a time series; 5, 10, 20, 40, and 80 min after L-valine addition, resulting in a total of eight samples (**Figure 1A**). A culture of spike-in *E. coli* cells was grown in a parallel, which was not exposed to L-valine (**Figure 1B**). The spike-in cells carry an inducible plasmid and express three transcripts that are not present in the wildtype strain, namely a *V. cholerae* sRNA (*qrr2*), an antibiotic marker (*bla*), and an antisense transcript of *lacI*, from the plasmid. The spike-in cells were induced with IPTG for approximately four generations before they were mixed with the experimental samples in a 1:100 ratio based on OD. We added spike-in cells to the experimental samples prior to total RNA extraction to ensure that variations in RNA recovery, cDNA synthesis, and sequencing depth between the samples would be reflected in the numbers of spike-in reads. By using this approach, we were able to, very precisely, quantify the relative changes in the transcriptome during the experiment, while we lost the information about absolute amounts of transcripts mapped, which is only obtainable by addition of *in vitro* transcribed spike-in RNA after sample preparation (Gorochowski et al., 2019).

### Overview of Spike-In Methodology and RNAseq Data

RNAseq libraries were prepared from the eight samples collected during the isoleucine starvation time series. Illumina sequencing results produced 22 to 29 million reads per sample and the proportion of uniquely mapped reads to the *E. coli* genome (RefSeq NC_U00096.3) was at least 97.8% for all samples. The reads from each sample were mapped in parallel to the spike-in plasmid reference sequence (**Supplementary Table S1**). The volume of spike-in cells added to each sample prior to RNA purification was adjusted according to the samples' OD at the time of harvest, to ensure a constant ratio of spike-in cells to sampled cell mass (as measured by OD). We first assessed the spike-in method by calculating the ratio of spike-in reads to total reads. Thus, we could evaluate two parameters; i), how much the three steady-state replicate samples varied from each other and ii), whether the spike-in method indicated changes in total RNA levels during starvation. As seen in **Supplementary Figure S1A**, the ratio of plasmid reads to total reads of the three replicates taken during steady-state growth varied only by ~1%, indicating a high reproducibility of the data. In contrast, as starvation progressed within the 80-min time series, the ratio of plasmid reads/total reads increased, indicative of a decline in total RNA levels from the experimental samples, which is consistent with the net negative effect of ppGpp on the activity of RNA polymerase (Fiil et al., 1972; Sarubbi et al., 1988) and break-down of rRNA (Zundel et al., 2009; Fessler et al., 2020) and tRNA (Svenningsen et al., 2017) upon starvation. However, the correlation deviated from the expected ratio at the 20-min time point with approximately 30% from the trend. The deviation is most likely due to erroneous sampling, which results in a surge in the ratio of ribosomal reads to spike-in reads at the 20-min time point (**Supplementary Figure S1B**). The surge in rRNA mid-starvation is highly unlikely to have a biological explanation, given the negative effect of amino acid starvation and ppGpp production on rRNA synthesis (Sands and Roberts, 1952; Cashel and Kalbacher, 1970). Therefore, we regarded the 20-min time point as an outlier and did not include it in the further analysis of the transcriptome. We then proceeded with normalizing the sequencing reads to the spike-in RNA (here designated RPKSP, Reads Per Kilobase of gene per 10 kilobase of spike-in, see *Materials and Methods*). For comparison we also normalized the sequencing reads (excluding reads mapping to the spike-in plasmid) using the standard method that only takes into account the sequencing depth and gene length (RPKM, Reads per Kilobase Million).

### Hallmark Stringent Response Gene Regulation Is Captured With RNAseq

**Figure 2:**
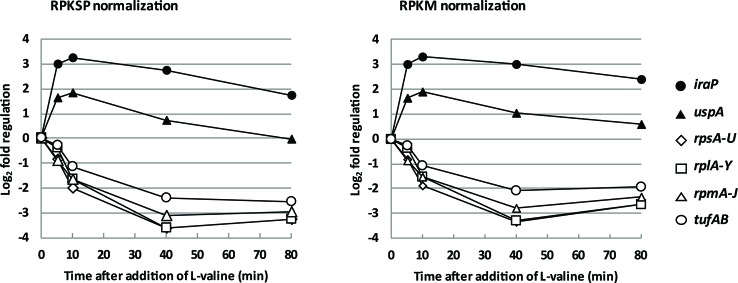
The messenger RNA (mRNA) encoding protein components of the protein synthesis machinery are rapidly down-regulated, and known ppGpp-controlled stress response proteins are rapidly up-regulated upon isoleucine starvation. The average levels of ribosomal protein mRNA reads during isoleucine starvation plotted as log_2_-fold change relative to pre-starvation levels (*rpsA-U*: open diamonds, *rplA-Y*: open squares, and *rpmA-J*: open triangles) as well as the average mRNA levels of elongation factor EF-Tu (*tufAB*: circles) and mRNA levels of the anti-adaptor protein, *iraP* (closed circles), and universal stress protein A, *uspA* (closed triangles).

When *E. coli* experiences amino acid starvation, transcription of the protein synthesis machinery is adjusted within minutes to meet the lower demand for protein synthesis (Maaløe, 1979; Ryals et al., 1982; Nomura et al., 1984). This hallmark of the stringent response was clearly reflected in our transcriptomic data, shown in **Figure 2** by the mRNAs encoding ribosomal proteins and elongation factor Tu. **Figure 2** also shows that two extensively characterized promoters known to be activated by ppGpp, namely the *iraP* and *uspA* promoters (Nyström and Neidhardt, 1992; Bougdour et al., 2006; Vollmer and Bark, 2018), are up-regulated in this analysis. Thus, the ppGpp-mediated stringent response is activated upon L-valine-induced isoleucine starvation in our experiment, and the general trends are detected using both methods of data normalization (RPKSP and RPKM).

### Ribosomal RNA Turnover Upon Isoleucine Starvation

**Figure 3:**
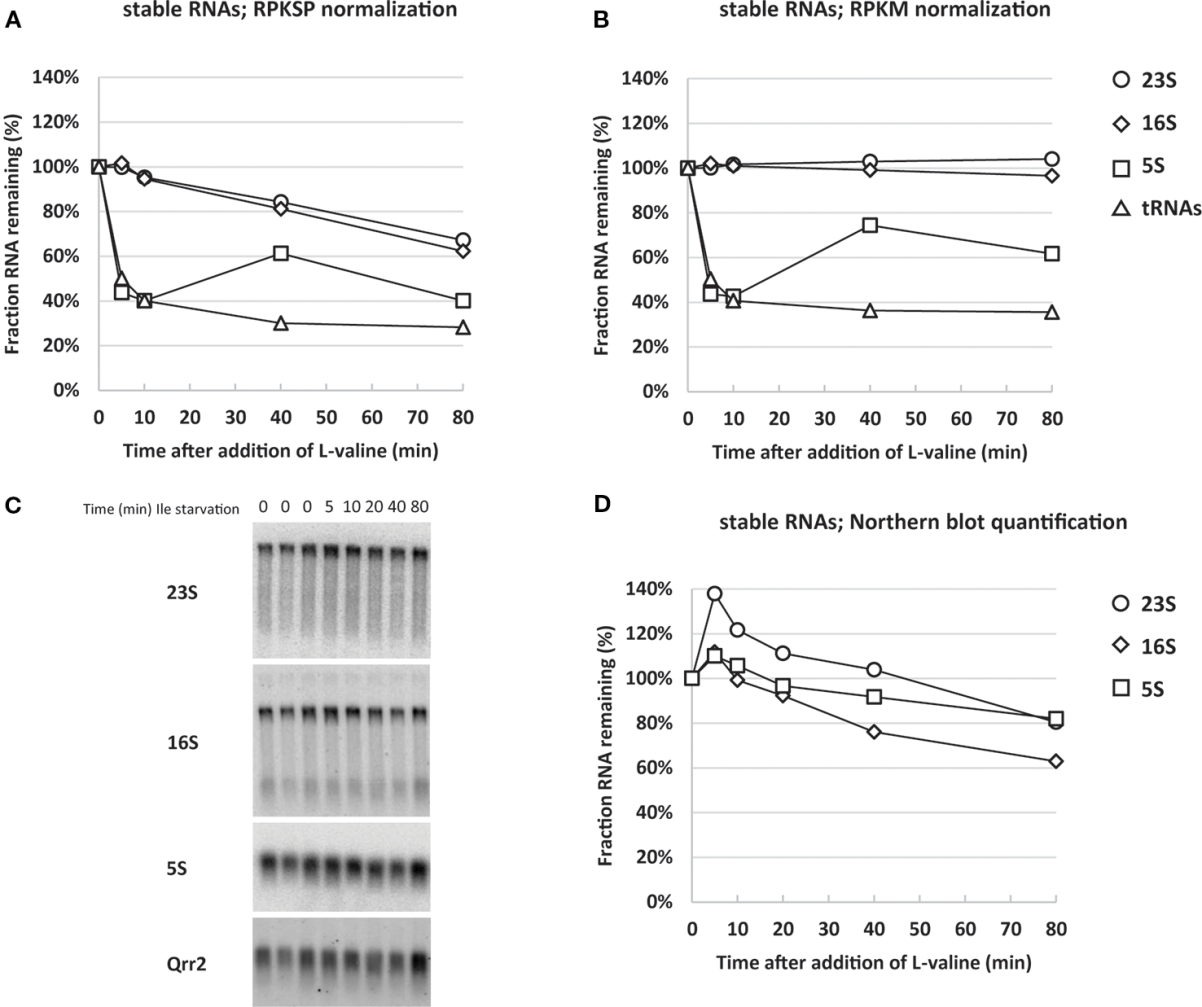
Normalizing RNA sequencing reads to spike-in RNA reveals that stable RNA levels are substantially reduced during isoleucine starvation. The average reads of stable RNA (23S; circles, 16S; diamonds, 5S; squares and transfer RNAs (tRNAs); triangles) during isoleucine starvation are shown relative to the average pre-starvation levels normalized with two different methods: **(A)** by spike-in cells (RPKSP, Reads Per Kilobase of gene per 10 kilobase of spike-in) and **(B)** by total reads (RPKM, Reads per Kilobase Million). **(C)** A 1% agarose gel was used for electrophoresis of total RNA from three samples harvested in steady-state growth before induction of isoleucine (Ile) starvation (0 time points) and during starvation (5, 10, 20, 40, 80 min time points). The resulting blot was probed for 23S, 16S, 5S, and the spike-in-cell-specific RNA Qrr2 as indicated. **(D)** The levels of stable RNA (23S; circles, 16S; diamonds, 5S; squares) were quantified by normalizing to Qrr2 from the spike-in cells and shown relative to the average of the three RNA samples harvested prior to starvation. The quantified and normalized data originates from the blot in panel **(C)**.

Assessing the ribosomal RNAs, however, our spike-in-normalized data show that not only was the synthesis of rRNA down-regulated, but the levels of existing rRNA per OD unit of cells were substantially reduced upon isoleucine starvation. Specifically, after 80 min of starvation the levels of 16S and 23S rRNAs had decreased to approximately 70% of the pre-starvation level (**Figure 3A**, RPKSP). This behavior was only visible when we normalized the sequencing reads to levels of reads from the spike-in plasmid, and not to total reads (**Figure 3B**, RPKM). In agreement with the RPKSP-normalized data, northern blots showed that 16S and 23S rRNAs decayed to approximately 60–80% of the pre-starvation level in the first 80 min after starvation (**Figures 3C, D**).

While there is good agreement between the two methods for 16S and 23S rRNA, there is a discrepancy in the quantification of 5S rRNA levels. In the northern blot analysis, 5S levels declined to approximately 80% after 80 min, whereas RNAseq reads indicate a decline to approximately 40% of the pre-starvation level. We suspect that the lower levels of 5S reads is likely a consequence of a higher number of mapping errors for short RNAs in the RNAseq pipeline, as we noticed a general increase in the variation between the triplicate steady-state samples for reads mapping to short genes (<0.2 kb) (**Supplementary Table S2**). As a further quantification control, we assessed the RNA samples by qRT-PCR for the levels of 5S. The qRT-PCR data verified the magnitude of 5S decline shown in the northern blots (**Supplementary Figure S2**), confirming that 5S was unreliably quantified in the RNAseq pipeline. Collectively, the RPKSP-normalized transcriptome, the northern blots, and the qRT-PCR assay, validate that rRNA levels decrease substantially during the early response to isoleucine starvation.

### Transfer RNA Turnover Upon Isoleucine Starvation

**Figure 4:**
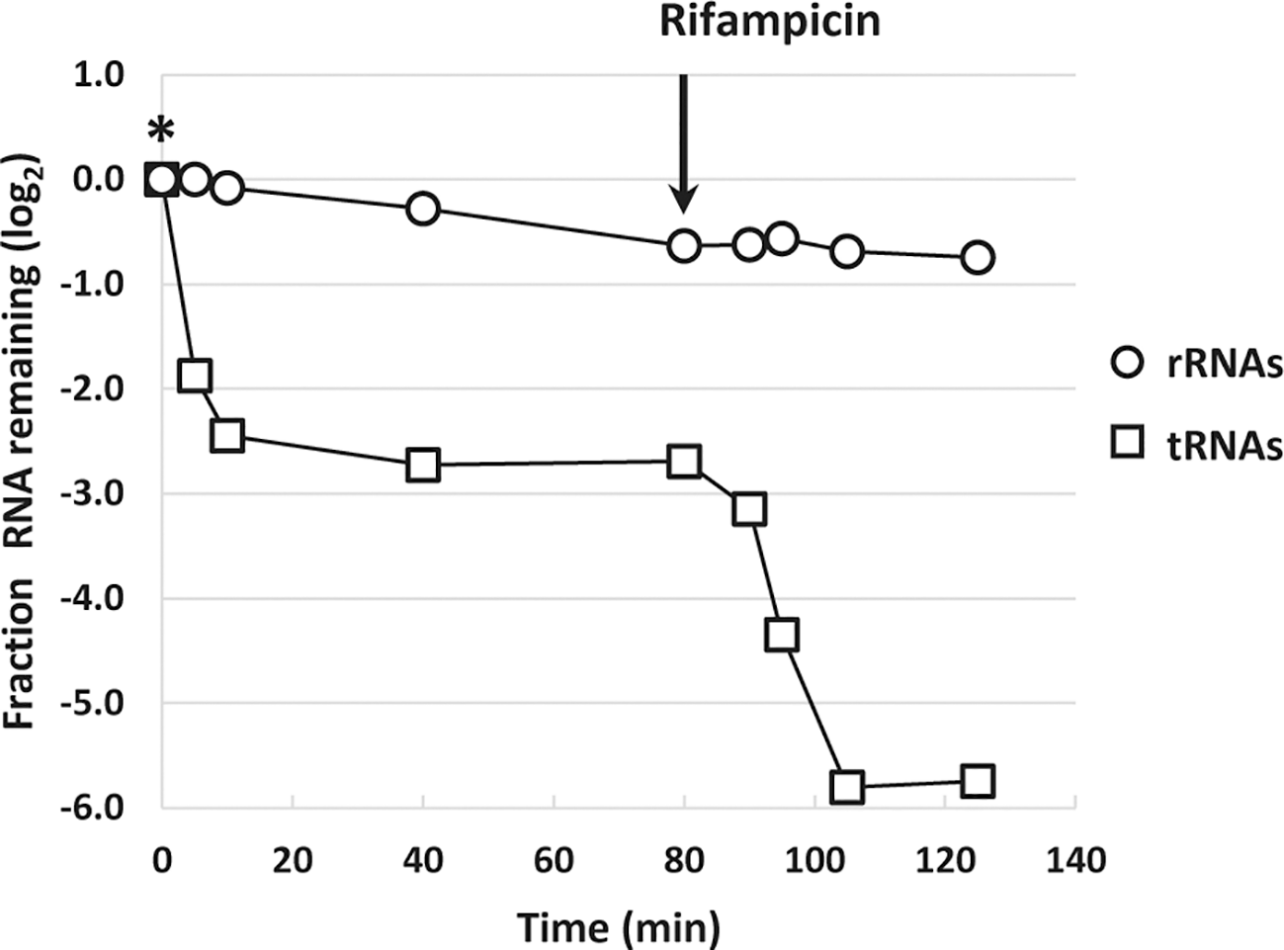
Inhibition of transcription leads to diminished levels of transfer RNA (tRNA)-mapped reads. The sum of reads mapped to ribosomal RNA (rRNA) genes (open circles) and the sum of reads mapped to tRNA genes (open squares) during isoleucine starvation are shown as the log_2_-fold change relative to their pre-starvation levels (L-valine addition denoted by *). Immediately after the last isoleucine starvation sample was harvested at 80 min, 300 μg/ml rifampicin was added to the culture to block transcription initiation. Samples were harvested 10, 15, 25, and 45 min after rifampicin addition.

A rapid reduction in tRNA levels upon L-valine-induced isoleucine starvation as well starvation for other amino acids was reported previously, but the kinetics of tRNA disappearance shown in **Figure 3** are much faster than expected from northern blot experiments (Svenningsen et al., 2017), regardless of the method of normalization. In addition, the concentration of tRNA is highly underestimated by the RNAseq method as a molar ratio of about 10 tRNAs per ribosome is expected (Dong et al., 1996), but we only detected 0.003 tRNA per rRNA by RNAseq during steady-state growth (**Supplementary Table S3**). The low detection of tRNAs is reportedly due to the difficulties in reverse transcription of the highly modified tRNA to cDNA (Motorin et al., 2007). While tRNA is quantified independently of its modification status in northern blots, here it is not, and we therefore expect newly transcribed hypomodified tRNA to be overrepresented in the RNAseq analysis. This could explain why tRNA “disappears” fast (within 5 min of the onset of starvation; **Figure 3**) as transcription of tRNA genes is curtailed by the stringent response, so the pool of hypomodified tRNA is expected to decrease very fast upon starvation and enter the pool of poorly detected mature tRNA. Indeed, treatment of the starved culture with the transcription initiation inhibitor rifampicin, which terminates initiation of RNA synthesis, resulted in an additional decrease in tRNA-mapped reads down to just 2% of the pre-starvation level, supporting that very little mature tRNA was detected by RNAseq (**Figure 4**), while previous northern blot experiments showed at least 20% retention of tRNA 80 min after rifampicin treatment (Svenningsen et al., 2017). By contrast, the profile of rRNA levels per OD unit of culture remained nearly undisturbed during the rifampicin treatment (**Figure 4**).

### Changes in the Size of the Total Messenger RNA Pool Upon Isoleucine Starvation

**Figure 5:**
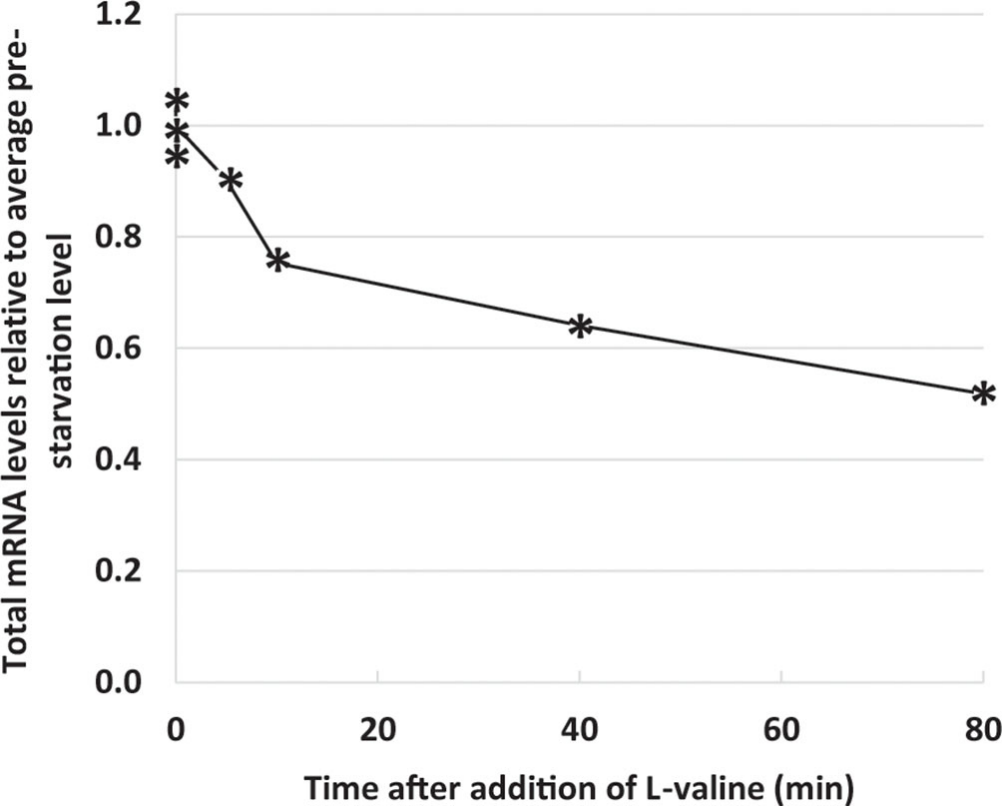
Total messenger RNA (mRNA) levels decrease during starvation. Reads mapping to 129 noncoding RNAs [ribosomal RNA (rRNA), transfer RNA (tRNA), small regulatory RNAs, and the RNA component of RNase P] were removed from the filtered **Supplementary Data Sheet 1** to yield the mRNA dataset (**Supplementary Table S4**). The sum of reads mapped to the 3.919 mRNA genes during isoleucine starvation are plotted relative to the average of their pre-starvation levels.

The whole-cell spike-in method in combination with RNAseq allowed us to estimate the kinetics of the reduction in the total mRNA pool during starvation (**Figure 5**). This estimate is unique in that it yields direct information on mRNA abundance per OD unit of bacterial culture under starvation relative to steady-state levels, whereas previous estimates were based on the change in synthesis rates of stable RNA relative to total RNA (R_S_/R_T_) during starvation (Ryals et al., 1982), or the addition of synthetic RNA spike-in after the preparation of sample RNA (see e.g., Schena et al., 1995; Gorochowski et al., 2019). It is well known that the promoter selectivity and the initiation frequency of RNA polymerase changes as a function of the ppGpp concentration (Kajitani and Ishihama, 1984; Sanchez-Vazquez et al., 2019) and that ppGpp switches RNA polymerase onto stress-related genes rather than genes for components of the translational apparatus (as illustrated in **Figure 2**). It has also been shown that the processivity of the RNA polymerase is negatively affected by the concentration of ppGpp (Kingston and Chamberlin, 1981; Kingston et al., 1981; Sørensen et al., 1994; Vogel and Jensen, 1994; Roghanian et al., 2015). In the present set of data (**Figure 5**) we can see how these effects of reduced RNA polymerase initiation frequency, processivity, and altered promoter selectivity combined to reduce the total mRNA pool to about 70% already after 10 min of starvation, and reduced it by half after 80 min (**Figure 5** **and** **Supplementary Table S4**).

In summary, the spike-in methodology allowed us to quantify the change in the pools of rRNA and mRNA upon isoleucine starvation and subsequent rifampicin treatment, while tRNA could not be reliably quantified using this method. Northern blot analysis confirmed the decrease in rRNA shown by RPKSP normalization (**Figure 3C**). The underlying reason that RPKSP reveals this decrease while RPKM normalization does not, is that since the rRNA comprises ~85% of total RNA in the cell (on average 89% of the total reads in our samples), a decrease in rRNA will result in an almost equivalent decrease in the total RNA. Therefore, a normalization method that relies on sequencing depth will i) mask changes in very abundant rRNAs, ii) underestimate the magnitude of the change in RNAs that change in the same direction as the very abundant RNA, and iii) overestimate the magnitude of the change in RNAs that change in the opposite direction of the very abundant RNA.

### Transcriptome-Wide Response to Amino Acid Starvation Induced by L-Valine

The sequencing results for individual genes are available in **Supplementary Data Sheet 1** (RPKSP-normalized) and **Supplementary Data Sheet 2** (RPKM-normalized) as the RNA abundance levels (normalized reads per gene) for each time point as well as the log_2_–fold difference in RNA abundance levels at each time point of starvation and rifampicin treatment, relative to the average of the three steady-state samples. **Supplementary Tables S5** and **S6** are alphabetic lists of RPKSP- and RPKM-normalized genes, that are up- or down-regulated more than two-fold at the 80-min starvation time point relative to the average of the three steady-state time points. Finally, **Supplementary Tables S8** and **S9** report the 100 RPKSP-normalized genes most strongly activated and repressed, respectively, upon isoleucine starvation.

**Figure 6:**
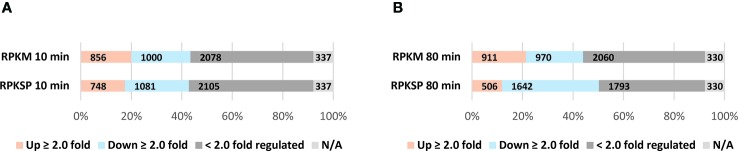
Applying the spike-in methodology alters the proportions of up- and down-regulated genes observed during isoleucine starvation. Normalization was done by two different methods; RPKM (Reads Per Kilobase Million) and RPKSP (Reads Per Kilobase of gene per 10 kilobase of spike-in). RNAs that showed >2-fold change at **(A)** 10 min and **(B)** 80 min after starvation relative to the average of the three samples harvested immediately before isoleucine starvation are denoted in orange bars (≥ 2.0 fold up-regulated) and blue bars (≥ 2.0 fold down regulated). Genes that were < 2.0-fold regulated genes are shown in dark gray bars. N/A: not included in the analysis either because the reads in the triplicate steady state samples were filtered out (see *Materials and Methods*), or gene reads were not detected in either steady-state growth or starvation (light gray bars).

The difference in the outcomes of the two normalization methods for L-valine-induced isoleucine starvation was assessed by plotting the 10 and 80 min time points relative to the average of the three steady-state samples (**Figures 6A, B**). We apply a two-fold regulatory threshold (Wren and Conway, 2006) to ease comparison between our data sets and the most relevant literature (Traxler et al., 2011; Sanchez-Vazquez et al., 2019). As shown in **Figure 6**, RPKM normalization underestimates the number of down-regulated genes in the stringent response compared to normalization to the spike-in reads (RPKSP). This effect is more pronounced as starvation progresses. RPKM normalization fails to detect 40% of the ≥ 2.0 fold down-regulated genes at 80 min post starvation, which are detected with RPKSP normalization (**Figure 6**). By contrast, RPKM overestimates the number of genes induced ≥2 fold by >40% at the 80 min time point, compared to RPKSP normalization (**Figure 6B**). While the number of genes that qualify for the ≥2 fold up- or down-regulation cut-off clearly differ substantially between the two normalization methods, we emphasize that the identity of the most strongly regulated genes is independent of the normalization method. Thus, the 970 genes that could be identified as ≥2-fold down-regulated in the RPKM-normalized data set despite the tendency for this method to overestimate gene expression late in starvation relative to the steady state, form the most strongly down-regulated subset of the 1642 genes that were identified as ≥2-fold down-regulated after application of the RPKSP correction, and *vice versa* for the up-regulated genes (**Supplementary Tables S5** and **S6**). From this result, it is evident that the method of normalization is critical for the interpretation of changes in RNA levels when cells experience a shift in growth condition. In the remainder of the manuscript, we therefore focus on the RPKSP-normalized data set as we analyze the transcriptomic response to amino acid starvation induced by L-valine.

### Two Temporal Profiles Account for the Majority of Gene Expression Changes

**Figure 7:**
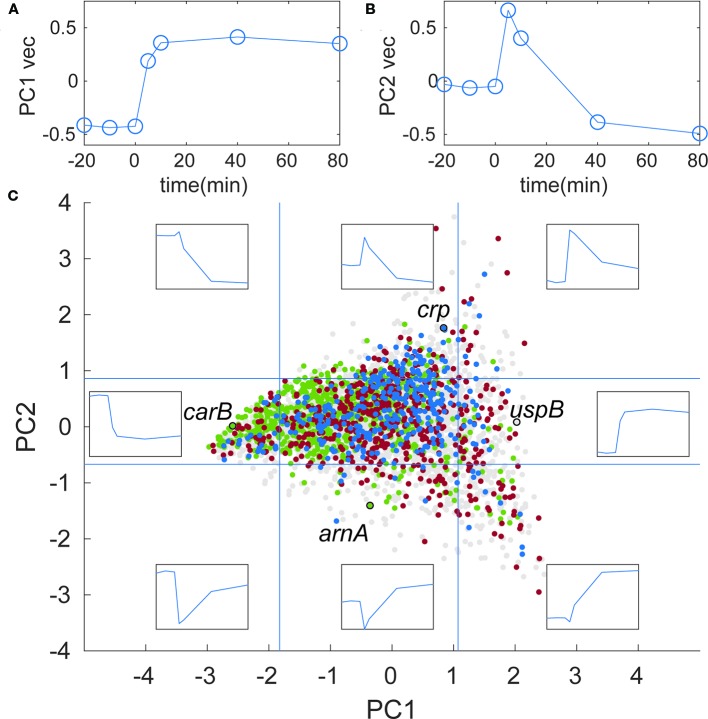
Two distinct temporal profiles account for the majority of gene expression changes in the stringent response transcriptome. **(A)** Temporal profile of the PC1 vector. The three steady-state samples are artificially displayed between time −20 and 0 to better illustrate the shape of the vector. Units on the y-axis are arbitrary units of normalized RNA levels, as described in *Materials and Methods*. **(B)** Temporal profile of the PC2 vector; **(C)** 4,048 genes plotted according to their PC1 and PC2 values. Inserts show temporal profiles at the corresponding coordinates, e.g., the top left insert shows the profile for PC1 = −3 and PC2 = 3. Vertical blue lines indicate the cut-off values for genes in the subset with 10% highest and lowest PC1 values used for enrichment analysis. Horizontal blue lines indicate the same for the PC2 values. Each data point corresponds to a gene and is colored according to the parent GO term it belongs to. Since some genes belonged to more than one category, the coloring was layered so that the final graph displays the smallest category the data point belongs to: all data points (4,048 points) light gray; biosynthetic process (975 points) green; response to stress (578 points) red; regulation of transcription, DNA-templated (349 points), blue. The points corresponding to the genes mentioned in the main text are highlighted with black open circles and labeled with the gene names.

**Figure 8:**
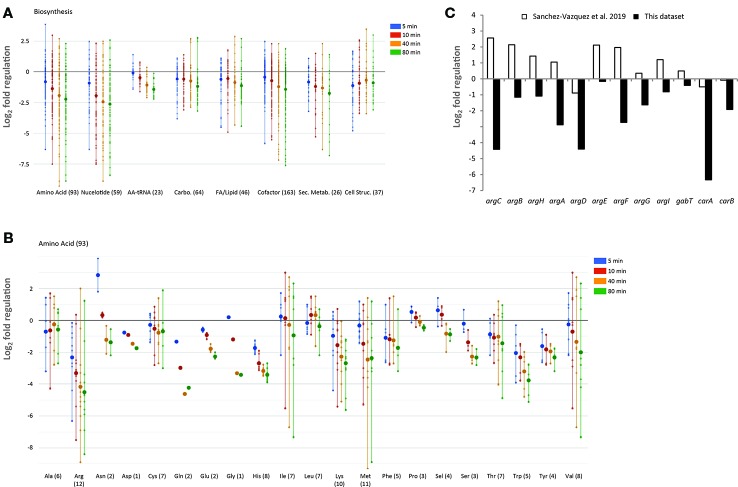
Regulation of biosynthetic genes upon L-valine-induced isoleucine starvation. **(A)** Temporal profile of biosynthetic genes as displayed by the “Omics Dashboard” tool (Paley et al., 2017). Each gene in a class is represented by a dot for each of the four time-points during starvation (5, 10, 40, and 80 min) The position on the y-axis indicates the log2-fold change in RNA level relative to the average of the three steady-state samples. The large circles represent the average change of genes in each category at each time point. Genes are classified as belonging to amino acid biosynthesis, nucleoside and nucleotide biosynthesis, carbohydrate biosynthesis, fatty acids and other lipids biosynthesis, aminoacyl-tRNA synthetases, biosynthesis of small molecules (co-factors, prosthetic groups, electron carriers, and vitamins) that participate in enzyme reactions, biosynthesis of secondary metabolites, and biosynthesis of cell structural elements (mainly cell wall). A gene can be assigned to more than one class. The number of genes in each class is indicated in parentheses. **(B)** Temporal profile of biosynthetic genes belonging to the sub-system amino acid biosynthesis. **(C)** Change in messenger RNA (mRNA) level of genes in the arginine biosynthesis pathway 5 min after addition of L-valine, relative to the average of the three steady-state samples (black bars). White bars show the relative change of the same mRNAs 5 min after ectopic induction of high levels of ppGpp produced by a constitutive variant of *relA* as reported by Sanchez-Vazquez et al. (2019).

To explore trends in the transcriptome response to isoleucine starvation in general terms, a principal component analysis (PCA) was carried out. PCA is a statistical procedure that uses linear transformations of the original data (relative abundance of each RNA at the seven time points, see *Materials and Methods*) to define a set of new, orthogonal variables that reduce the number of variables needed to describe the data set. We found that the first two components of the analysis account for 69% of the variability of the data. The temporal profile of these two components (PC1 and PC2) are shown in**Figures 7A, B** respectively, and PC1 and PC2 scores for each individual gene is provided in **Supplementary Table S7**. The PC1 vector, which accounts for 48% of the variability, describes genes that do not show variation among the three steady-state samples, change abruptly in response to the addition of L-valine, and remain at the new level throughout the duration of the starvation. An example of a gene with a high positive PC1 score is *uspB*, encoding the universal stress protein B, which is known to be induced by starvation (Farewell et al., 1998). Like PC1, the PC2 vector describes genes that do not vary among the three steady-state samples, and change abruptly in response to the addition of L-valine. But in contrast to PC1, the RNA level for genes with a high positive PC2 score show a surge at the 5 min time-point followed by a drop as starvation continues. An example of such a gene is *crp*, encoding the cAMP-binding global transcriptional regulator CRP. **Figure 7C** shows all 4,048 genes in the transcriptome analysis plotted according to their PC1 and PC2 scores (colored dots). The temporal profiles for selected values of PC1 and PC2 are shown as eight inserts at the corresponding positions on the graph. For example, the insert at position (−4;0) depicts a gene with a temporal profile that is strongly negatively correlated with the PC1 profile shown in **Figure 7A**. An example of such a gene is *carB*, encoding a component of carbamoyl phosphate synthetase, which is involved in arginine biosynthesis from ornithine (see also **Figure 8C**). The gene *arnA* encoding a key enzyme in outer membrane lipid A modification (Williams et al., 2005), is an example of a gene with a negative correlation to the PC2 profile. Specifically, *arnA* mRNA was quite abundant during steady-state growth, dropped 15-fold at 5 minutes after starvation, and returned to steady-state levels after 40 min of starvation. The temporal profile of *uspB*, *crp*, *carB*, and *arnA* were confirmed by qRT-PCR and show similar relative expression profiles as the RPKSP-normalized sequencing data (**Supplementary Figure S3**).

To explore whether general trends could be discerned in terms of the biological processes associated with temporal profiles defined in the PCA, we used the enrichment tool available on the EcoCyc webserver (Keseler et al., 2017) to identify gene ontology (GO) terms that were statistically over-represented in the four subsets of genes that scored among the 10% highest or lowest for PC1 or PC2 (see *Materials and Methods*). Among the tens to hundreds of GO terms that were significantly enriched in each subset, we focused on broad categories (parent GO terms) to highlight the general trends in the dataset, rather than focus on specific metabolic pathways or regulons. The 10% of genes that had the highest PC1 scores were enriched (p-value 3*10^−7^) for the parent GO term “response to stress” (**Figure 7C**, red dots). By contrast, the 10% of genes that had the lowest PC1 scores were highly enriched (p-value 7*10^−13^) for the broad GO term “biosynthetic process” (**Figure 7C**, green dots). Meanwhile, the 10% of genes that had the highest PC2 scores were enriched (p-value 2*10^−4^) for “regulation of transcription, DNA-templated” (**Figure 7C**, blue dots), while those with the 10% lowest PC2 scores did not yield a significantly enriched broad category. While there are naturally many outliers within these broad categories, this analysis illustrates that general temporal profiles can be recognized in the stringent response that distinguish biosynthesis genes which generally remain down-regulated during starvation (low PC1), and stress response genes which generally remain up-regulated (high PC1), from the transcriptional regulators whose expression spikes during the growth transition followed by a recovery period (high PC2).

### Downregulation of Biosynthesis

The “Omics Dashboard” software tool available on the EcoCyc webserver (Paley et al., 2017) was used to further explore the transcriptomic changes that occurred in response to L-valine addition. The tool combines data on the expression level of individual genes into a hierarchy of cellular systems and subsystems. As expected in response to amino acid starvation, and as indicated in the PCA analysis, genes responsible for the major biosynthetic processes (e.g., nucleotide, carbohydrate, fatty acid, lipid, and aminoacyl-tRNA synthesis) are generally down-regulated (**Figure 8A**). Amino acid biosynthesis genes are reportedly up-regulated under the stringent response, which could help *E. coli* overcome starvation (Cashel et al., 1996), and this has been observed to varying extents in previous transcriptome-wide analyses (Cashel et al., 1996; Durfee et al., 2008; Traxler et al., 2008; Sanchez-Vazquez et al., 2019). We found that amino acid biosynthetic genes were generally down-regulated in response to L-valine (**Figure 8B**). For example, while seven of the 12 genes ascribed to arginine biosynthesis were up-regulated within 5 min of ppGpp production in a recent study where a constitutively active RelA variant was induced to produce ppGpp in the absence of starvation (Sanchez-Vazquez et al., 2019), none of the arginine biosynthetic genes were up-regulated in our study (**Figure 8C**). We expect that the key difference between the two experiments is that all amino acids were supplied in the growth medium in the study conducted by Sanchez-Vazquez and coworkers, so at the outset of the experiment, arginine biosynthetic operons would be repressed by the arginine-bound ArgR repressor (Caldara et al., 2006). What is then measured is a positive regulatory effect of ppGpp on some of these repressed promoters. In our study, the growth medium did not contain any amino acids prior to the addition of L-valine, so the amino acid biosynthetic operons were already de-repressed when ppGpp production was induced by L-valine-mediated isoleucine starvation. We suspect that the reduced rate of protein synthesis that occurs upon isoleucine starvation results in a build-up of the residual amino acids, including arginine, which would lead to repression of the arginine biosynthesis pathway by arginine-bound ArgR.

### Differential Response of the RpoS and Lrp Regulons

**Figure 9:**
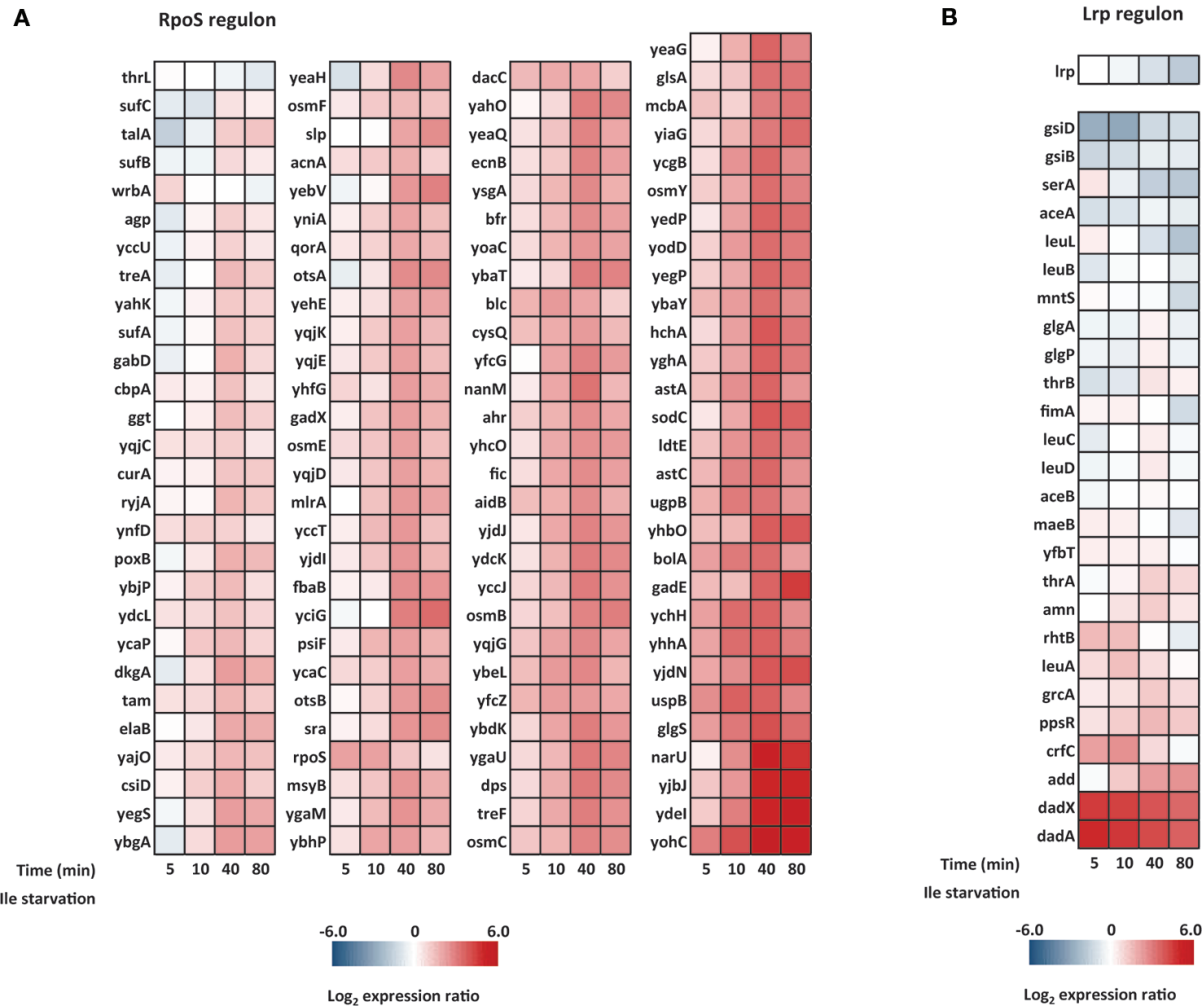
Heat maps of the RpoS and Lrp regulons in cells starved for isoleucine. The time points into isoleucine starvation is indicated below each heat map. **(A)** The ppGpp/RpoS regulon and **(B)** the ppGpp/Lrp regulon. Genes listed in each regulon were defined in a previous study by Traxler and coworkers (Traxler et al., 2011).

In most bacterial systems, the stringent response includes a robust general stress response mediated by the stationary phase sigma factor RpoS (σ^S^ or σ^38^), which is regulated at the level of transcription, translation as well as at the level of protein stability both directly and indirectly by ppGpp (Lange and Hengge-Aronis, 1994; Landini et al., 2014). The RpoS regulon has been studied extensively and is known to control >140 genes in response to diverse stress conditions (Lacour and Landini, 2004; Weber et al., 2005), including isoleucine starvation (Traxler et al., 2011). In the study by Traxler and coworkers, they analogously applied isoleucine starvation on the conditional auxotrophic *E. coli* K-12 strain, but contrasting our experiments, the cells in their experimental set-up gradually exhausted isoleucine in media containing all other amino acids. They show that the levels of ppGpp calibrate and co-regulate the RpoS-dependent stress response and the Lrp-dependent regulon (leucine responsive protein), which mostly includes genes for metabolic enzymes. The Lrp-dependent response occurred prior to and at lower ppGpp concentrations than the RpoS-dependent response (Traxler et al., 2011). We employed the definitions of the RpoS and Lrp regulons used by Traxler et al., and investigated the isoleucine starvation response of these two regulons in our experimental set-up. As seen in **Figure 9A**, the majority of the RpoS-dependent genes are induced after 40 min of L-valine-mediated isoleucine starvation, in agreement with the slow but robust induction of the regulon reported in the previous study. However, under the condition tested here, *E. coli* did not significantly induce the Lrp regulon apart from genes involved in alanine metabolism (*dadAX*) (**Figure 9B**). In fact, the *lrp* mRNA itself was three-fold down-regulated at the end-point of the starvation. In line with this finding, the small regulatory RNA GcvB was among the top 10 up-regulated transcripts in our experiment (**Supplementary Table S8**), and GcvB is known to regulate the *lrp* mRNA negatively (Holmqvist et al., 2012; Lee and Gottesman, 2016; Lalaouna et al., 2019). It is unknown to us how valine-induced isoleucine starvation could trigger high expression levels of GcvB but we suggest that the induction of GcvB could be the main reason for the missing Lrp response in this particular experimental set-up.

### A Specific Transcriptional Response to L-Valine-Induced Isoleucine Starvation

**Figure 10:**
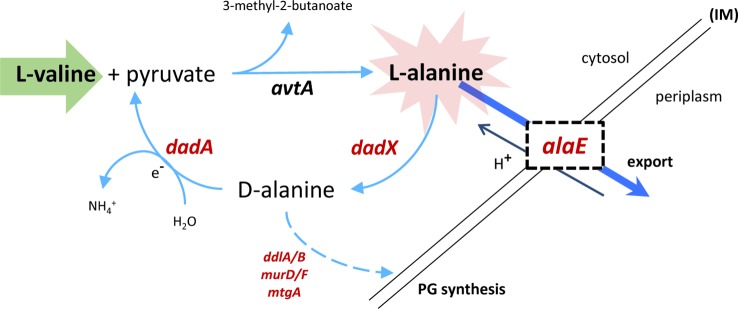
Export pathway for excess L-valine. The synthesis of L-alanine requires pyruvate and is catalyzed by the valine-pyruvate aminotransferase *avtA*. The predominant alanine racemase in the cell, *dadX*, is degradative and catalyzes the conversion of L-alanine to D-alanine. The respiratory-chain-associated *dadA* can further catabolite D-alanine to pyruvate which can enter the central metabolism, or in this case be used as substrate to further convert high concentrations of L-valine to L-alanine. *alaE*, an alanine-proton antiporter, facilitates the export of L-alanine out from the cytosol in exchange of a proton. *ddlA/B, murD/F,* and *mtgA* are all involved in biosynthesis of cell wall structural elements. PG, peptidoglycan; IM, inner membrane). Up-regulated genes in the pathway are represented in red.

The only gene which was up-regulated more strongly than GcvB 5 min after starvation was *alaE*, encoding an L-alanine exporter (Hori et al., 2011), which showed an average increase of transcript levels during starvation >300-fold compared to pre-starvation levels (**Supplementary Table S8**). *alaE* transcription was recently shown to be positively regulated by ppGpp (Sanchez-Vazquez et al., 2019), supporting the up-regulation seen here. Although not included in the Lrp regulon defined in (Traxler et al., 2011), *alaE* is also predicted to be up-regulated by Lrp. Similarly, *dadA* and *dadX* which are identified here as the only clearly up-regulated genes in the curtailed Lrp-regulon (**Figure 9B**) were identified to be transcriptionally activated by ppGpp in the same study (Sanchez-Vazquez et al., 2019). We suggest that the prominent up-regulation of *alaE, dadA, dadX,* and *gcvB* results directly from cellular metabolic consequences of the addition of L-valine rather than the resulting starvation for isoleucine. The physiological role of AlaE is to export L-alanine (and possibly alanine dipeptide) to avoid intracellular toxic-level accumulation of L-alanine (Kim et al., 2015). The excess L-valine, together with pyruvate as substrate, can be converted to L-alanine by *avtA,* one of three major alanine-synthesizing transaminases in *E. coli* (Hori et al., 2011). The *avtA* mRNA was not up-regulated during starvation, but it was highly expressed during unrestricted growth under our conditions (**Supplementary Data Sheet 1**), suggesting that the transaminase protein it encodes is abundant at the onset of starvation. According to this model, overabundant levels of L-alanine is exported out from the cell by AlaE. In addition, surplus L-alanine can be converted by the alanine racemase *dadX* to D-alanine (Wild et al., 1985), which in turn is the substrate for the D-amino acid dehydrogenase *dadA* in the inner membrane to yield ammonium and pyruvate (Franklin and Venables, 1976). This further fuels the conversion of L-valine to L-alanine (**Figure 10**). Evidently, excess L-valine gave rise to high levels of L-alanine that is countermeasured by upregulating the mRNA encoding the alanine exporter, clearly envisaged in this transcriptome. Moreover, elevated levels of D-alanine in the cells might be utilized and directed to cell wall synthesis. Some cell structure biosynthetic genes were de-repressed as starvation progressed (**Figure 8A**), especially genes involved in UDP-MurNAc-pentapeptide biosynthesis (e.g., *ddlB* and *murD/F*) and peptidoglycan maturation (*mtgA*) (**Supplementary Table S10**).

## Discussion

The stringent response to amino acid starvation is in many respects a model system for studies of bacterial stress responses, and has been the subject of intense study for decades, including several transcriptome-wide studies (Durfee et al., 2008; Traxler et al., 2008; Traxler et al., 2011). Here, we combined RNAseq with spike-in-cell normalization of the sequencing depth to obtain an adjusted view of the stringent response that is independent of any assumptions about the total RNA content of the cells. The methodology allowed us to quantify the changes in total rRNA and total mRNA per OD unit of bacterial culture over the first 80 min of starvation for isoleucine. In accordance with other reports (Ben-Hamida and Schlessinger, 1966; Jacobson and Gillespie, 1968; Maruyama and Mizuno, 1970; Zundel et al., 2009; Piir et al., 2011; Fessler et al., 2020), we find that the stability of rRNA is compromised upon nutrient starvation, resulting in a drop in rRNA per OD unit to 70% of the pre-starvation level within the first 80 min of an amino acid starvation. Because rRNA constitutes the vast majority of cellular RNA, this drop affects the quantification of all other RNA species in the cell if the RNAseq data is normalized solely to the sequencing depth of the samples in the conventional way (referred to here as RPKM-normalization, **Figure 6**). One important outcome of our work is therefore that ~40% more mRNAs are down-regulated, and ~40% fewer are up-regulated by more than two-fold, compared to what a conventional RNAseq study would suggest. We remark that the problem associated with normalizing solely to sequencing depth is not solved by depletion of rRNA prior to RNA-seq, because the rRNA-depleted transcriptome also is subject to the transcriptional consequences of a change in growth conditions. For example, the activity of RNA polymerase is reduced at elevated ppGpp levels, giving rise to lower RNA levels and the RNA chain growth rate is decreased (Kingston and Chamberlin, 1981; Kingston et al., 1981; Sørensen et al., 1994; Vogel and Jensen, 1994; Roghanian et al., 2015).

This study highlights that although the stringent response of *E. coli* to amino acid starvation has a set of defining characteristics, most notably a surge in ppGpp levels and reduced transcription of genes encoding the protein synthesis machinery, the particular growth conditions employed give notable differences in the transcriptome-wide response at the detailed resolution of an RNAseq experiment. Most notably, in contrast to a previous study (Traxler et al., 2011) the extensive Lrp regulon was not activated in response to isoleucine limitation in this study, and amino acid biosynthesis was not generally induced although many operons encoding amino acid biosynthesis genes are activated in response to ppGpp under other growth conditions (Sanchez-Vazquez et al., 2019). We used principal component analysis combined with enrichment analyses to identify broad functional classes of genes that responded similarly to the growth transition. Besides these, the data set also contains many smaller gene categories that will be of interest to specific research sub fields. For example, mRNA of the conserved BluR-repressed operon *ycgZ-ymgABC*, which were completely repressed during steady-state growth, were among the genes most strongly up-regulated upon starvation (**Supplementary Table S8**), suggesting an unidentified regulatory mechanism that is unrelated to the known BluR signals; blue light and low temperature, for the YcgZ regulator of OmpF porin expression and the Ymg biofilm modulators (Tschowri et al., 2012; Duval et al., 2017). Finally, at least four (*alaE*, *gcvB*, *dadA*, *dadX*) of the 20 genes that respond most strongly in our dataset are likely responding to the sudden addition of L-valine rather than the starvation for isoleucine (**Figure 10**).

The methodology also allowed us to quantify changes in total mRNA levels under starvation (**Figure 5**), and the result underlines that overall mRNA production is substantially reduced upon amino acid starvation. The rapid reduction of the total mRNA pool demonstrated here supports the model previously proposed to explain why the initial surge in ppGpp levels upon amino acid starvation levels off on a timescale of a few minutes, namely that the initial surge of ppGpp in response to the onset of starvation should taper off due to a reduction in the number of RelA-associated stalled ribosomes, resulting from the reduced availability of mRNA (Sørensen et al., 1994; Tian et al., 2016).

## Data Availability Statement

The raw reads generated for this study have been deposited in NCBI's Gene Expression Omnibus with the accession ID: GSE136753.

## Author Contributions

BG, MS, and SLS conceived and designed the study. BG, AB, MF performed experiments. BG, SAS, NM, MS, and SLS analyzed the data. BG, MS, and SLS wrote the paper.

## Funding

We acknowledge funding of this project by the Danish National Research Foundation (DNRF120) and the Independent Research Fund Denmark (8049-00071B and 8021-00280A).

## Conflict of Interest

The authors declare that the research was conducted in the absence of any commercial or financial relationships that could be construed as a potential conflict of interest.

## Footnotes

1 Andrews, S. FastQC: a quality control tool for high throughput sequence data (http://www.bioinformatics.babraham.ac.uk/projects/fastqc/).

2 (https://www.ncbi.nlm.nih.gov/geo/query/acc.cgi?acc=GSE136753)
