# Supplementary material for: Valine-Induced Isoleucine Starvation in Escherichia coli K-12 Studied by Spike-In Normalized RNA Sequencing
Source: Front Genet. 2020 Mar 5;11:144. doi: 10.3389/fgene.2020.00144 (PMC7066862; doi:10.3389/fgene.2020.00144)
Supplement: Supplementary file 4 [file Presentation_1.pdf]

Supplementary Figure S1

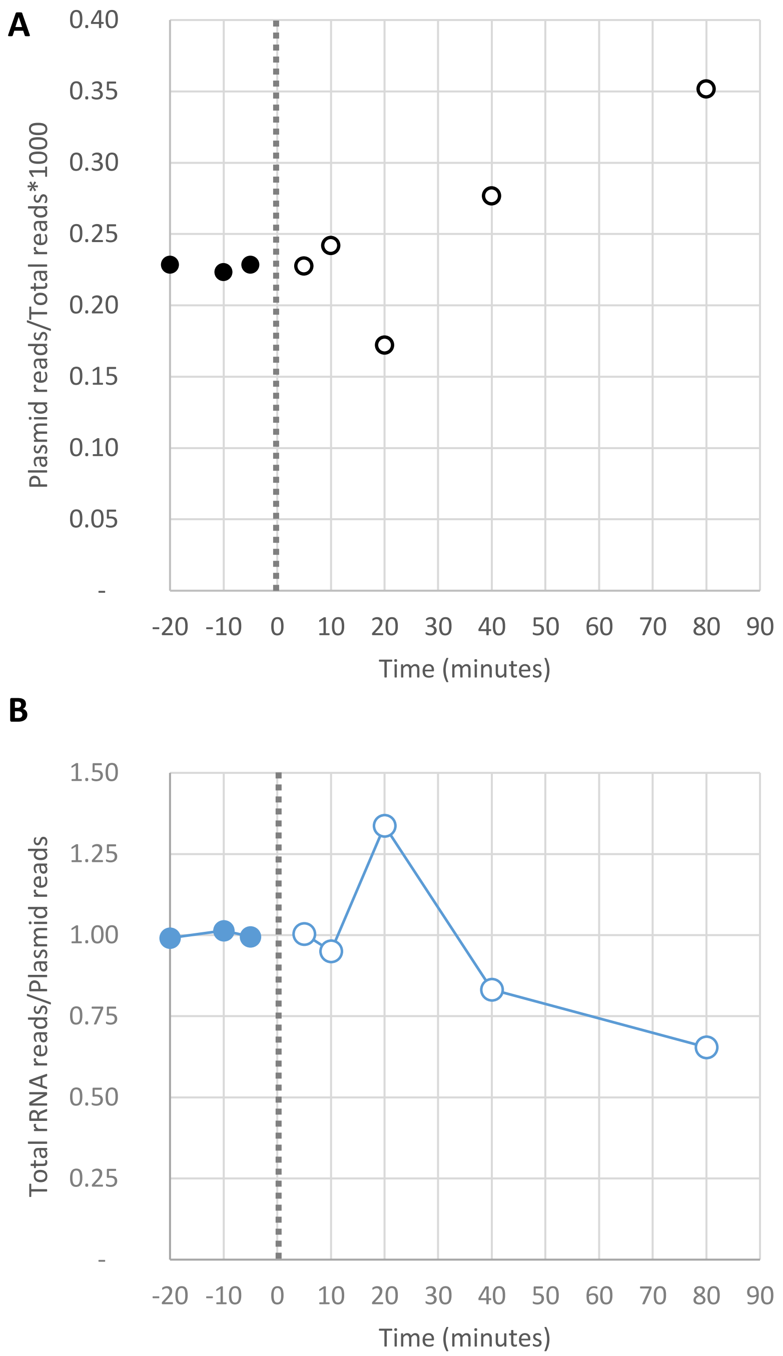

Figure S1. Total RNA levels are reduced upon isoleucine starvation. A) The raw spike-in reads (plasmid reads) is shown as a fraction of the total reads in three steady state samples (closed circles) and five starvation samples (open circles). **B)** The raw rRNA reads is shown as a fraction of spike-in reads (plasmid reads) in three steady state samples (closed circles) and five starvation samples (open circles). Each data point is shown relative to the average of the three steady-state samples. Time of induction of isoleucine starvation is shown as a vertical dashed line. The three steady-state samples are artificially displayed between time -20 and -5.

Supplementary Figure S2

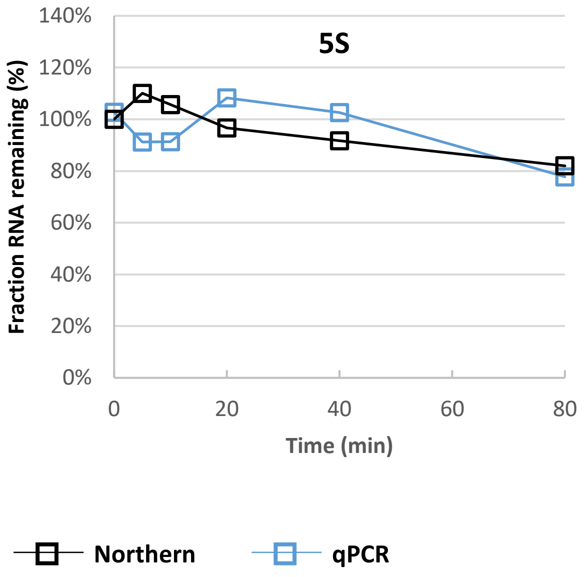

Figure S2. Quantification of 5S by northern blot verified with quantitative PCR (qPCR). The levels of 5S were assessed by qPCR and compared to northern blot data. RNA levels are shown relative to the average of the three RNA samples harvested prior to starvation. The quantified and normalized northern blot data originates from the blot in panel 3C in the main text.

Supplementary Figure S3

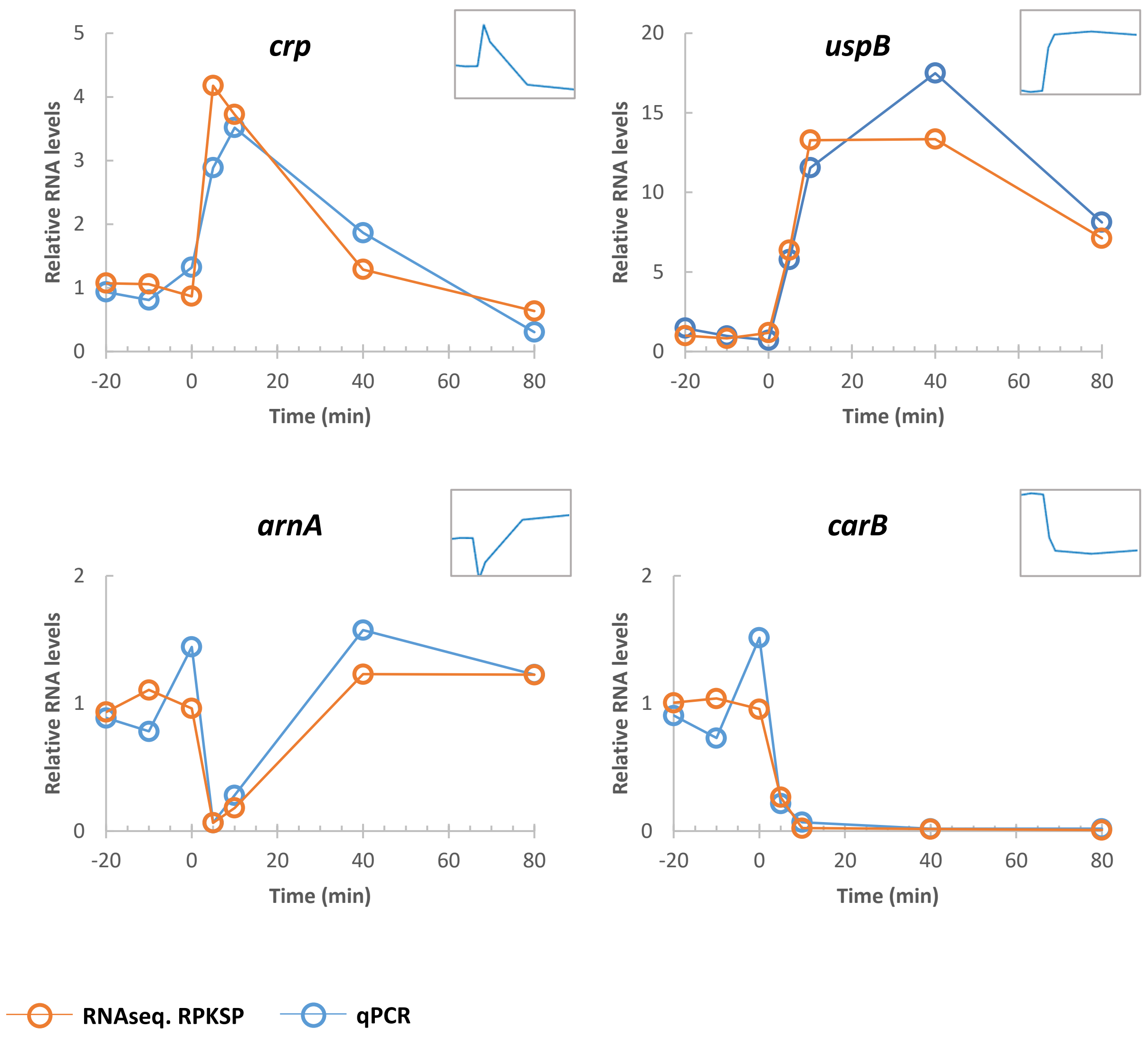

Figure S3. RPKSP-normalized RNA sequencing reads verified with quantitative PCR (qPCR). Four genes; *crp, uspB, arnA* and *carB,* were assessed by qPCR and compared to RPKSP normalized sequencing data. The relative RNA levels in the three steady state samples before induction of isoleucine (Ile) starvation are artificially displayed between time -20 and 0 followed by 5, 10, 40 and 80 min starvation time points. Inserts show temporal profiles of the corresponding subcategory were the gene belongs according to the principal component analysis shown in **Figure 7C** in the main text.
